# rs1944919 on chromosome 11q23.1 and its effector genes *COLCA1*/*COLCA2* confer susceptibility to primary biliary cholangitis

**DOI:** 10.1038/s41598-021-84042-x

**Published:** 2021-02-25

**Authors:** Yuki Hitomi, Yoshihiro Aiba, Yosuke Kawai, Kaname Kojima, Kazuko Ueno, Nao Nishida, Minae Kawashima, Olivier Gervais, Seik-Soon Khor, Masao Nagasaki, Katsushi Tokunaga, Minoru Nakamura, Makoto Tsuiji

**Affiliations:** 1 Department of Microbiology, Hoshi University School of Pharmacy and Pharmaceutical Sciences, 2-4-41 Ebara, Shinagawa-ku, Tokyo, 142-8501 Japan; 2 Clinical Research Center, National Hospital Organization (NHO) Nagasaki Medical Center, Omura, Japan; 3 Genome Medical Science Project, National Center for Global Health and Medicine, Tokyo, Japan; 4 Tohoku Medical Megabank Organization, Tohoku University, Sendai, Japan; 5 The Research Center for Hepatitis and Immunology, National Center for Global Health and Medicine, Ichikawa, Japan; 6 Japan Science and Technology Agency (JST), Tokyo, Japan; 7 Human Biosciences Unit for the Top Global Course Center for the Promotion of Interdisciplinary Education and Research, Kyoto University, Kyoto, Japan; 8 Department of Hepatology, Nagasaki University Graduate School of Biomedical Sciences, Omura, Japan; 9 Headquarters of PBC Research in NHO Study Group for Liver Disease in Japan (NHOSLJ), Clinical Research Center, NHO Nagasaki Medical Center, Omura, Japan

**Keywords:** Immunogenetics, Primary biliary cirrhosis, Genome-wide association studies

## Abstract

Primary biliary cholangitis (PBC) is a chronic, progressive cholestatic liver disease in which intrahepatic bile ducts are destroyed by an autoimmune reaction. Our previous genome-wide association study (GWAS) identified chromosome 11q23.1 as a susceptibility gene locus for PBC in the Japanese population. Here, high-density association mapping based on single nucleotide polymorphism (SNP) imputation and in silico/in vitro functional analyses identified rs1944919 as the primary functional variant. Expression-quantitative trait loci analyses showed that the PBC susceptibility allele of rs1944919 was significantly associated with increased *COLCA1*/*COLCA2* expression levels. Additionally, the effects of rs1944919 on *COLCA1*/*COLCA2* expression levels were confirmed using genotype knock-in versions of cell lines constructed using the CRISPR/Cas9 system and differed between rs1944919-G/G clones and -T/T clones. To our knowledge, this is the first study to demonstrate the contribution of *COLCA1/COLCA2* to PBC susceptibility.

## Introduction

Primary biliary cholangitis (PBC) is a chronic, progressive cholestatic liver disease in which intrahepatic small bile ducts are destroyed. PBC is considered an organ-specific autoimmune disease for the following reasons: (1) existence of autoreactive T and B cells from PBC patients and well-defined autoantigens such as the E2 component of the pyruvate dehydrogenase complex, (2) high frequencies of complications of other autoimmune diseases, (3) overlap of many disease susceptibility gene loci with those of other autoimmune diseases, and (4) an overwhelming female predominance^1–5^. Both adaptive immune responses (including CD4^+^ T cells, CD8^+^ T cells, and B cells) and innate immune responses [including natural killer (NK) cells] mediate destruction of small bile ducts via reactions against biliary epithelial cells^5–7^.

Previous family-based studies ascertained the strong involvement of genetic factors in PBC development^8,9^. Recently, *human leukocyte antigen* (*HLA*) and 30 non-*HLA* loci were associated with PBC susceptibility in individuals of European descent through genome-wide association studies (GWASs), ImmunoChip analyses, and subsequent meta-analyses^10–17^. Additionally, our previous GWAS identified 8 susceptibility loci for PBC in the Japanese population^18–20^. 17 loci were also identified as susceptibility loci for PBC in the Chinese population by another group^21^. These results indicate that the genetic background related to antigen presentation and T cell-/B cell-mediated inflammation plays an important role in PBC development.

Candidate genes with well-known functions that are located near top-hit disease susceptibility single nucleotide polymorphisms (SNPs) are often selected as disease susceptibility genes in GWASs. However, the contribution of susceptibility loci to pathogenesis can be understood by the identification of effector genes regulated by primary functional variants located in disease susceptibility loci.

Here, effector gene and primary functional variant in a PBC susceptibility gene locus chromosome 11q23.1 were identified in the present study. In order to analyze the disease susceptibility of all genetic variation including the primary functional variant in this locus, we carried out high-density association mapping of chromosome 11q23.1 based on SNP imputation using data from a whole-genome sequence reference panel of 1070 Japanese individuals and our previous GWAS^18,19,22^. We then carried out in silico and in vitro functional analyses to identify primary functional variants. Finally, we attempted to identify effector genes and elucidate the molecular mechanism through which functional variants confer PBC susceptibility using expression-quantitative trait locus (e-QTL) analyses and gene editing using the CRISPR/Cas9 system.

## Results

### SNP imputation and high-density association mapping

In our previous GWAS using the Affymetrix Axiom Genome-Wide ASI1 array, only 11 SNPs exhibited *P*-values < 0.001 for PBC susceptibility at the chromosome 11q23.1 locus^18,19^. Therefore, to perform high-density association mapping for PBC susceptibility using all SNPs on chromosome 11q23.1, SNP imputation was performed using genotype data from our previous GWAS and a phased whole-genome sequencing reference panel of 1070 Japanese individuals [1KJPN; Tohoku Medical Megabank Organization, Tohoku University, Japan]^18,19,22^. After SNP imputation, 143 SNPs exhibited *P*-values < 0.001 for PBC susceptibility at this locus.

In addition to the SNPs that were installed on the Affymetrix Axiom Genome-Wide ASI1 array, 39 SNPs exhibited *P*-values < 5.0 × 10^–6^ by SNP imputation, probably due to linkage disequilibrium (LD) at this locus. Among these SNPs, 29 were located in the 5′ intergenic region of *POU class 2 associating factor 1* (*POU2AF1*), and 13 were located in the first intron of *POU2AF1*; however, no SNPs were located in exons or splice site (Table 1). Therefore, disease susceptibility in this locus appears to be associated with genetic variation affecting gene expression levels.

**Table 1:** SNPs associated with susceptibility to PBC in the Japanese population in chromosome 11q23.1 by high-density association mapping.

SNP_ID^a^	GWAS or Imputation^b^	Position (Chr.11)^c^	*P*^d^	OR^e^	UCSC^f^	Location
rs4938534	GWAS	111275133	1.25E−08	1.35	×	5′ of *POU2AF1*
rs12362038	Imputation	111270882	1.42E−08	1.35	×	5′ of *POU2AF1*
rs10891259	Imputation	111273686	1.93E−08	1.35	×	5′ of *POU2AF1*
rs4938541	Imputation	111280208	2.87E−08	1.34	△	5′ of *POU2AF1*
rs7952176	Imputation	111270228	3.78E−08	1.34	△	5′ of *POU2AF1*
rs1944926	Imputation	111287287	4.52E−08	1.34	△	5′ of *POU2AF1*
rs1123066	Imputation	111269435	6.17E−08	1.33	×	5′ of *POU2AF1*
rs4936432	Imputation	111269243	1.02E−07	1.33	×	5′ of *POU2AF1*
rs7952497	Imputation	111270281	1.23E−07	1.33	○	5′ of *POU2AF1*
rs4938518	GWAS	111267394	1.57E−07	1.32	×	5′ of *POU2AF1*
rs1944927	Imputation	111287404	3.41E−07	1.32	△	5′ of *POU2AF1*
rs10891261	Imputation	111276085	7.46E−07	1.31	×	5′ of *POU2AF1*
rs6589227	Imputation	111249367	1.39E−06	1.30	○	*POU2AF1* intron 1
rs1944919	GWAS	111259876	1.64E−06	0.78	○	5′ of *POU2AF1*
rs11213871	Imputation	111261112	1.67E−06	1.29	×	5′ of *POU2AF1*
rs1806294	Imputation	111264941	1.73E−06	0.78	△	5′ of *POU2AF1*
rs6589226	Imputation	111249226	1.80E−06	1.30	○	*POU2AF1* intron 1
rs1806397	GWAS	111264915	1.83E−06	0.78	△	5′ of *POU2AF1*
rs7947229	Imputation	111289354	1.93E−06	1.29	△	5′ of *POU2AF1*
rs4393359	Imputation	111294127	1.93E−06	1.30	×	5′ of *POU2AF1*
rs4489781	Imputation	111248177	2.01E−06	1.30	△	*POU2AF1* intron 1
rs6589224	Imputation	111246832	2.17E−06	1.29	×	*POU2AF1* intron 1
rs12799202	Imputation	111244756	2.48E−06	1.29	×	*POU2AF1* intron 1
rs12799471	Imputation	111244633	2.75E−06	1.29	×	*POU2AF1* intron 1
rs4622303	Imputation	111248514	2.76E−06	1.29	×	*POU2AF1* intron 1
rs4938508	Imputation	111245343	2.84E−06	1.29	×	*POU2AF1* intron 1
rs4938510	Imputation	111251476	2.85E−06	1.29	×	5′ of *POU2AF1*
rs10891264	Imputation	111293399	2.98E−06	0.78	×	5′ of *POU2AF1*
rs34563638	Imputation	111253801	3.22E−06	1.29	×	5′ of *POU2AF1*
rs4245182	Imputation	111245074	3.31E−06	1.29	×	*POU2AF1* intron 1
rs1944918	Imputation	111261995	3.53E−06	0.78	×	5′ of *POU2AF1*
rs4245183	Imputation	111245186	3.65E−06	1.29	×	*POU2AF1* intron 1
rs12293898	Imputation	111257068	3.70E−06	1.29	×	5′ of *POU2AF1*
rs7947717	Imputation	111289738	3.90E−06	1.29	×	5′ of *POU2AF1*
rs4356268	Imputation	111250650	3.94E−06	1.29	○	5′ of *POU2AF1*
rs12800418	Imputation	111257069	3.95E−06	1.29	×	5′ of *POU2AF1*
rs7116862	Imputation	111238440	4.02E−06	1.28	△	*POU2AF1* intron 1
rs6589225	Imputation	111246861	4.02E−06	1.29	×	*POU2AF1* intron 1
rs4529910	Imputation	111243102	4.13E−06	1.28	△	*POU2AF1* intron 1
rs3802843	Imputation	111250214	4.19E−06	1.28	-	5′ of *POU2AF1*
rs7946785	Imputation	111305419	4.52E−06	1.29	×	5′ of *POU2AF1*
rs35646619	Imputation	111295769	5.00E−06	1.28	×	5′ of *POU2AF1*

^a^SNPs with underlines were the final candidate primary functional variants.

^b^Genotyped by our previous GWAS (Kawashima M et al*.* 2017) or the imputed genotypes by the high-density association mapping in the present study.

^c^Position of the SNPs in hg19.

^d^*P* values calculated by Pearson's Chi-square test for the allelic model.

^e^Odds ratio (OR) of minor allele from the two-by-two allele frequency table.

^f^Probability of the functional damages checked by UCSC genome browser.

### Identification of the primary functional variant in chromosome 11q23.1

From 42 SNPs on chromosome 11q23.1 exhibiting *P*-values < 5.0 × 10^–6^, 5 SNPs (rs7952497, rs6589227, rs1944919, rs6589226, and rs4356268) (Table 1) located in DNase I hypersensitivity clusters and histone acetylation regions such as H3K27Ac in at least one cell type in the UCSC genome browser were selected as candidates conferring PBC susceptibility^23^. Additionally, the most significant SNP on chromosome 11q23.1 identified from high-density association mapping (rs4938534) (Table 1) was also selected as a susceptibility-conferring candidate.

**Figure 1:**
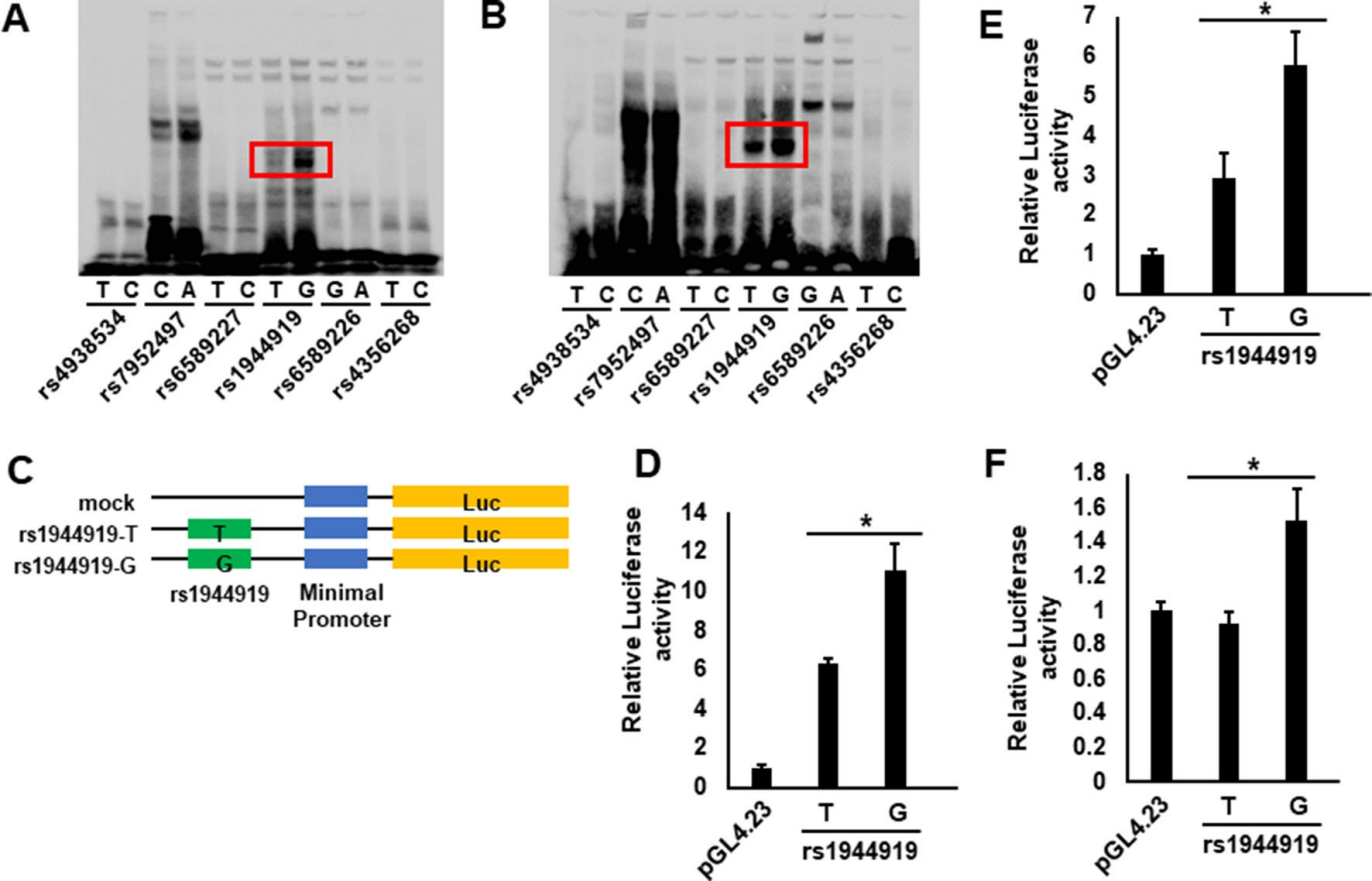
Identification of primary functional variants from candidate SNPs. (**A,B**) EMSA of each of the six candidate SNPs using biotin-labeled probes corresponding to major and minor alleles and nuclear extracts of Jurkat (**A**) and HepG2 (**B**) cells. rs1944919 was the only variant to exhibit a difference in mobility between the two alleles. (**C**) Outline of reporter plasmid constructs. PCR fragments containing rs1944919 were sub-cloned into the pGL4.23 vector. (**D–F**) Transcription was measured by cellular luciferase activity 24 h after transfection of Jurkat (**D**), HepG2 (**E**), and Raji (**F**) cells. The luciferase activity of cells transfected with the PBC susceptibility allele (G allele) of rs1944919 was increased compared with that of cells transfected with the T allele. Three independent experiments with triplicate measurements were performed for each assay. Data represent the mean ± SD; * *P* < 0.05 (Student’s *t* test).

EMSAs were performed to evaluate the effect of candidate SNPs on the binding affinity of transcription factors. Among candidate SNPs, a shift in mobility between major and minor alleles was detected for rs1944919 in Jurkat cells (human T lymphocytes) (Fig. 1A) and HepG2 cells (human liver carcinoma cells) (Fig. 1B). However, no differences in mobility were observed for the other candidate SNPs (rs4938534, rs7952497, rs6589227, rs6589226, and rs4356268) (Fig. 1A,B).

To assess differences in transcription efficiency between major and minor alleles of candidate SNPs, luciferase reporter assays were performed in HepG2, Jurkat, and Raji cells. In all of cell lines, the luciferase activity 24 h after transfection with a reporter construct containing the G allele (i.e., the PBC susceptibility allele^19^) of rs1944919 was significantly higher than that after transfection with a reporter construct containing the T allele (Fig. 1D–F). However, concordant with EMSA results, no differences in luciferase activity were observed for the other candidate SNPs (Supplementary Fig. 2). These results indicated that the primary functional variant on chromosome 11q23.1 was rs1944919.

**Figure 2:**
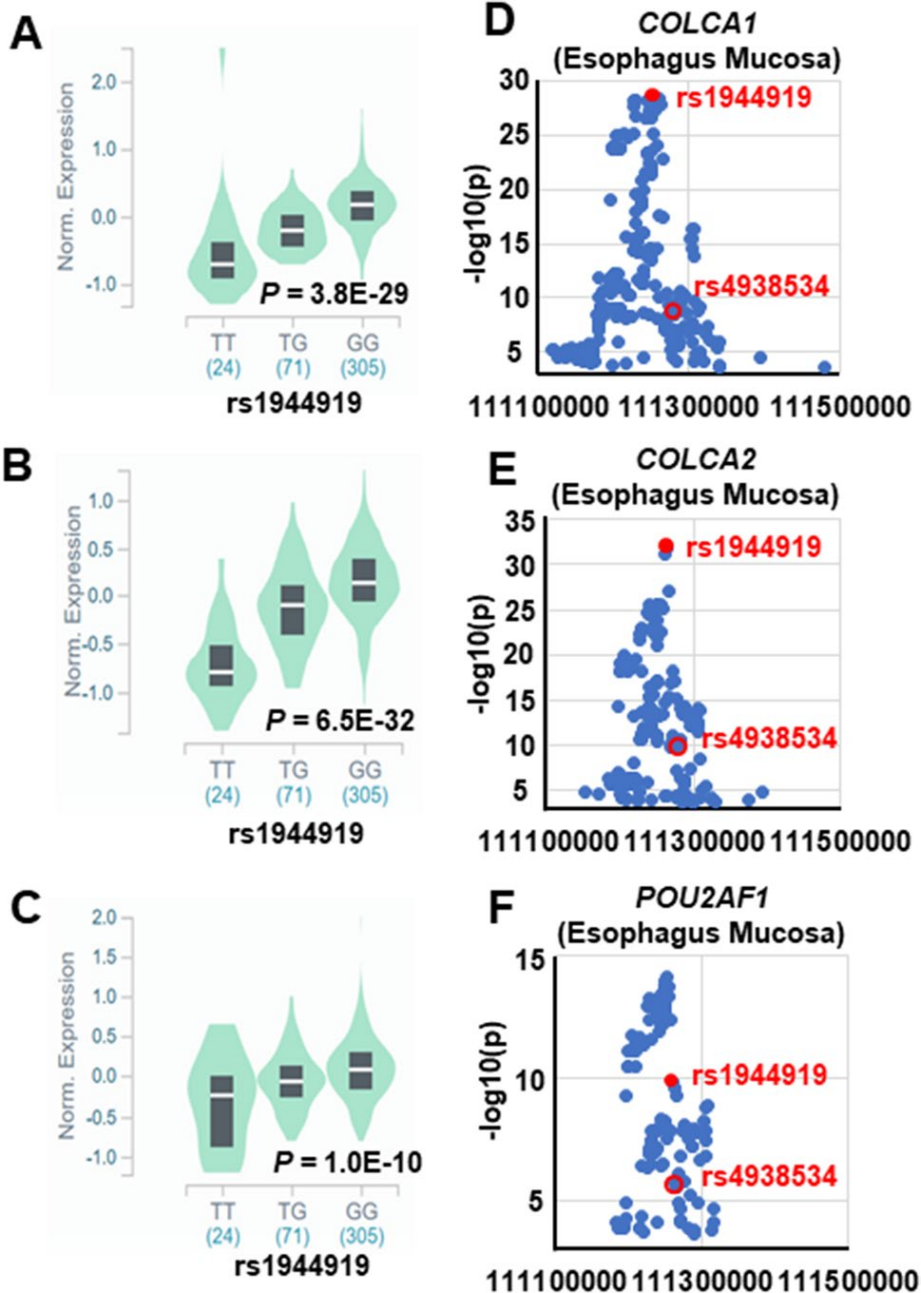
rs1944919 genotypes are associated with differences in endogenous *COLCA1*, *COLCA2*, and *POU2AF1* expression levels. (**A–C**) Effect of the rs1944919 genotype on endogenous expression of *COLCA1* (**A**), *COLCA2* (**B**), and *POU2AF1* (**C**) in the esophageal mucosa. (**D–F**) e-QTL mapping of *COLCA1* (**D**), *COLCA2* (**E**), and *POU2AF1* (**F**) in the esophageal mucosa. rs1944919, shown as red solid dots, exhibited the strongest association with *COLCA1* and *COLCA2* expression. Red open dots indicate rs4938534 which was the most significant SNP with PBC susceptibility on chromosome 11q23.1.

Consistent with luciferase assay results, the G allele of rs1944919, but not the T allele, was predicted to constitute a binding motif for several transcription factors according to the TRANSFAC database (Supplementary Fig. 1A and 1B)^24^. Among these transcription factors, only TATA box-binding protein associated factor 2 (TAF2) and E1A-binding protein P300 (EP300) were abundantly expressed in both Jurkat and HepG2 cells in GeneCard analyses (Supplementary Fig. 1C and 1D)^25^. However, the mobility of the G allele of rs1944919 was not super-shifted by preincubation with anti-TAF2 antibody or anti-EP300 antibody prior to electrophoresis in the EMSA (data not shown). These results suggest that other unknown transcription factors bind to the G allele of rs1944919.

### Identification of effector genes of rs1944919

In order to assess the effect of rs1944919 on gene expression, endogenous expression levels of all genes in the human genome in 47 organs and 2 transformed cell lines derived from healthy individuals were compared using the GTEx portal database^26^. Different rs1944919 genotypes significantly affected the expression of *colorectal cancer-associated 1* (*COLCA1*; *P* = 3.8 × 10^–29^), *COLCA2* (*P* = 6.5 × 10^–32^), and *POU2AF1* (*P* = 1.0 × 10^–10^) in the esophageal mucosa (Fig. 2A–C).

Although the difference of e-QTL was observed only in the esophageal mucosa, whole blood is the mixture of several immune cell subsets, and the EBV-transformed cell line is an cancer cell line. Therefore, we checked endogenous expression levels in each immune cell subset. Among immune cell subsets (i.e., CD4^+^ and CD8^+^ T cells, B cells, NK cells, and monocytes), significant associations between *COLCA1* and *COLCA2* expression levels in B cells and rs1944919 genotypes were observed (*COLCA1*: *P* = 0.00023; *COLCA2*: *P* = 0.00040)^27^. Consistent with luciferase assay results, higher expression levels were observed in individuals with the G allele compared to individuals with the T allele.

**Figure 3:**
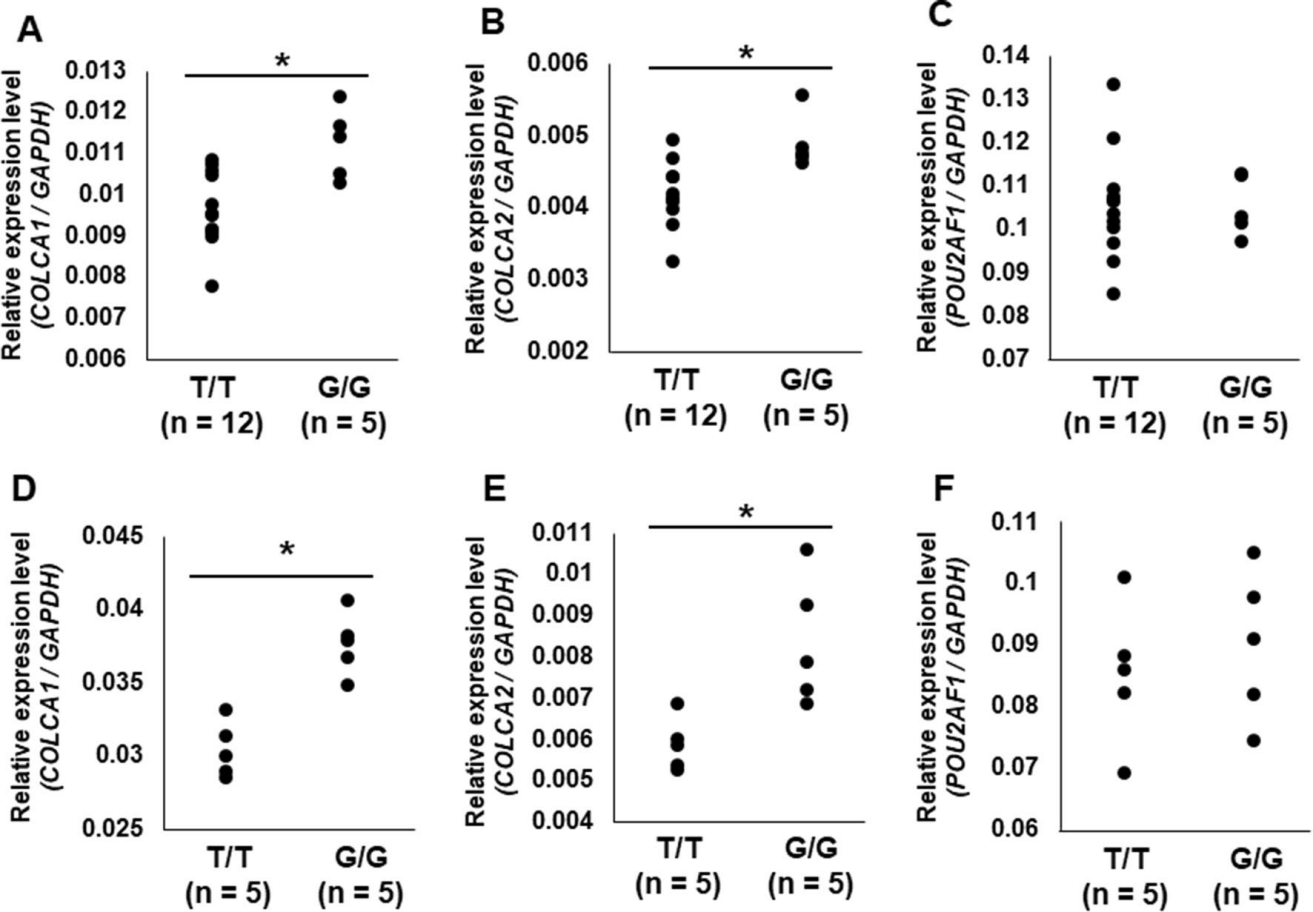
Effects of rs1944919 on gene expression in knock-in clones generated using the CRISPR/Cas9 system. Expression levels of *COLCA1* (**A**), *COLCA2* (**B**), and *POU2AF1* (**C**) in rs1944919-G/G and rs1944919-T/T knock-in clones of the Raji cell line. Expression levels of *COLCA1* (**D**), *COLCA2* (**E**), and *POU2AF1* (**F**) in rs1944919-G/G and rs1944919-T/T knock-in clones of the Jurkat cell line. *P < 0.01 (Mann–Whitney U test).

**Figure 4:**
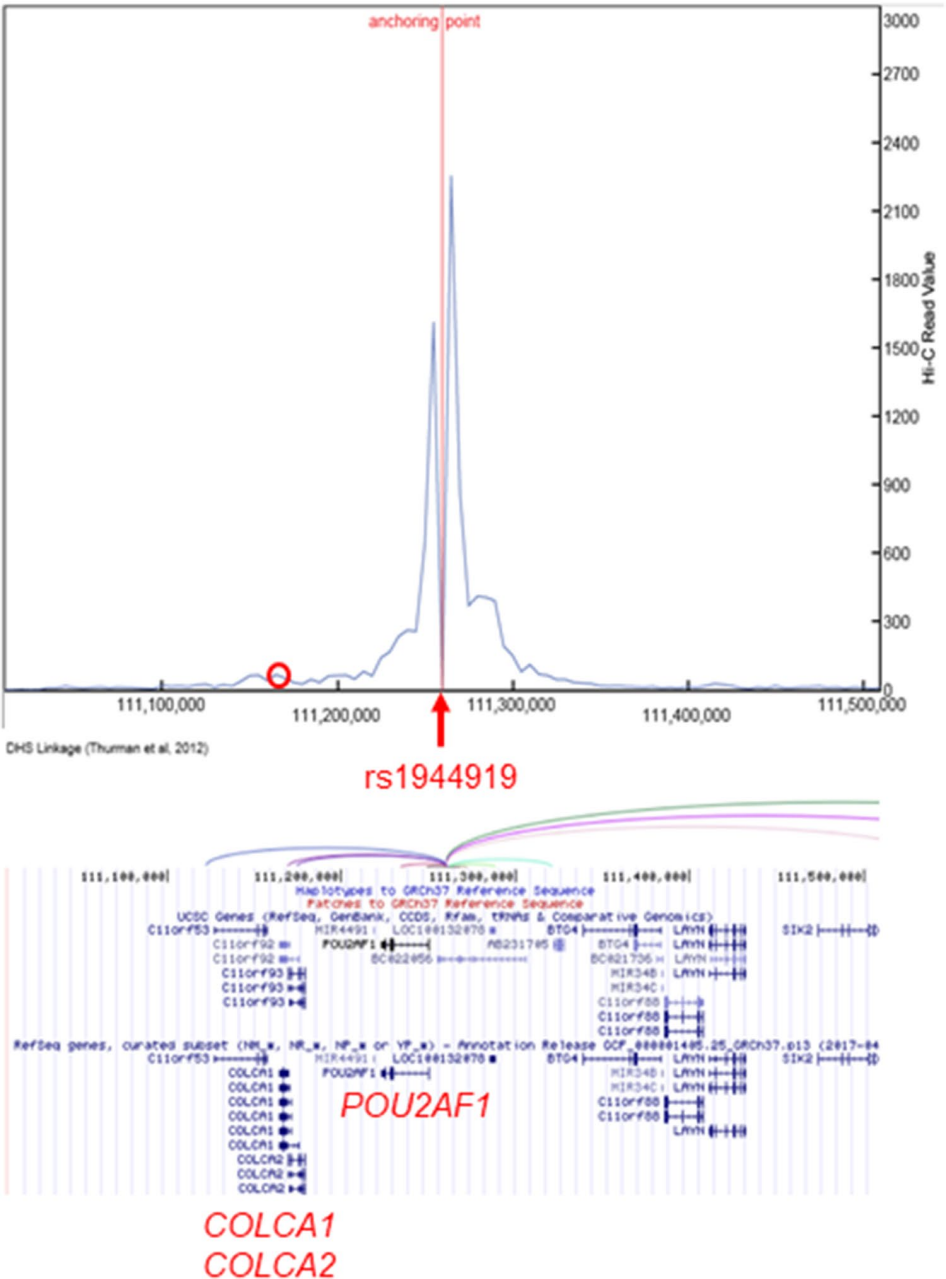
Chromatin interactions between the 5-kb window containing rs1944919 and upstream sequences of *COLCA* genes. Vertical line indicates the strength of interaction, which is denoted by a red circle. Interaction of the DNase high-sensitivity site (DHS; Thurman et al. 2012) also indicated an interaction between the two regions.

In general, due to LD, both primary functional variants and other genetic variants are associated with gene expression levels in e-QTL analyses. Therefore, the effect of rs1944919 on gene expression levels was assessed using rs1944919 genotype knock-in versions of Raji (human B lymphocytes) and Jurkat cell lines constructed using the CRISPR/Cas9 system. Among candidate effector genes, *COLCA1* and *COLCA2* were differentially expressed between rs1944919-T/T and -G/G clones (*P* < 0.01; Mann–Whitney U test) [Fig. 3A,B (Raji); Fig. 3D,E (Jurkat)]. Similar to the result of genome editing using the CRISPR/Cas9 system, rs1944919 exhibited the strongest associations with *COLCA1* and *COLCA2* expression levels among all genetic variations in the human genome by e-QTL association mapping analysis with the GTEx portal database (Fig. 2D,E)^26^. Additionally, despite a distance of 100 kb between the *COLCA* genes and rs1944919, chromatin interactions between the 5-kb window that contains rs1944919 and upstream sequences of the *COLCA* genes were detected in GM12878 cells (a line of transformed B cells isolated from a Caucasian individual) (Fig. 4)^28^. These results suggest that *COLCA1* and *COLCA2* are effector genes of rs1944919.

In contrast, although *POU2AF1* also exhibited a stronger association with rs1944919 than most of the other genetic variations, rs1944919 genotype knock-in versions of Raji and Jurkat cell lines did not show differences in *POU2AF1* expression level between rs1944919-T/T clones and -G/G clones [Fig. 3C (Raji) and 3F (Jurkat)]. Additionally, rs1944919 was not the top hit of *POU2AF1* expression level by e-QTL association mapping analysis (top-hit SNP: rs4356268) (Fig. 2F). Similar to e-QTL analysis, although splicing QTL (s-QTL) analysis of rs1944919 showed a significant association with *POU2AF1*, rs1944919 was not the top hit of *POU2AF1* splicing by s-QTL association mapping analysis (Supplementary Fig. 3).

Therefore, *POU2AF1* expression might be regulated by other genetic variations located near *POU2AF1*.

## Discussion

In the present study, although rs1944919 was located near *POU2AF1*, this variant affects the expression of other genes such as *COLCA1* and *COLCA2*, which are located 100 kb upstream from the SNP. In contrast to gene expression promoter regions that are located upstream of the transcription start site, expression enhancer regions are sometimes located more than 100 kb from the regulated genes. For example, rs12946510, which is located near *IKZF3* on chromosome 17q12-21, regulates the expression of *ORMDL3* and *GSDMB*, which are 200 kb away from rs12946510 at that locus^29^. Therefore, our approach for identifying the primary functional variant and effector gene consisting of high-density association mapping using SNP imputation analysis and subsequent in silico/in vitro functional analyses could overcome the difficulties of genetic analysis that are caused by the complicated DNA second structure.

**Figure 5:**
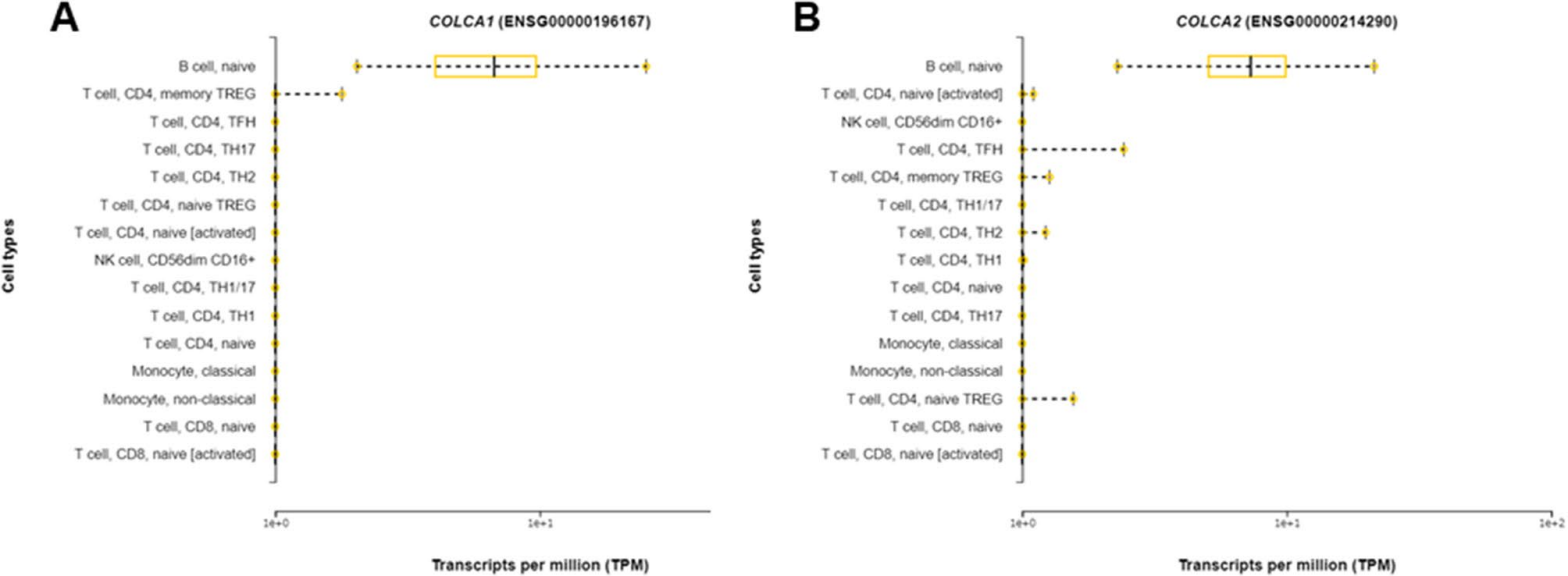
B cell-specific expression of *COLCA1* and *COLCA2* among lymphocytes. Expression levels of *COLCA1* (**A**) and *COLCA2* (**B**) in B cells, T cells, NK cells, and monocytes.

*COLCA1* and *COLCA2* were initially identified as genes whose expression levels are significantly associated with colorectal cancer (CRC) susceptibility SNP by Peltekova et al.^30^. In that report, characteristic differences in the histological pattern of lymphocyte infiltration in the lamina propria of the colon tissue were shown to be associated with *COLCA1* and *COLCA2* expression. Although the functions of COLCA1 and COLCA2 have yet to be fully elucidated, they are specifically expressed in human B cells among lymphocytes (Fig. 5)^31^**.** Mice harboring a mutation in *Colca2* exhibit increased numbers of leukocytes^32,33^. These results suggested that COLCA1 and COLCA2 might be involved in PBC development via the contribution to generate pathological B cell clones. This is the first study to indicate the possible contribution of COLCA1 and COLCA2 to PBC development. Further investigation of COLCA1 and COLCA2 as targets for PBC treatment is warranted.

rs4938534 is a SNP located at the 5′ end of *POU2AF1* and exhibited the most significant association with PBC susceptibility in the Japanese population in our previous GWAS^18,19^. POU2AF1 (also known as OBF-1, BOB-1, or OCA-B) is a transcriptional cofactor of Oct-1 (also known as POU2F1) and Oct-2 (also known as POU2F2), particularly in immunoglobulin (Ig)-secreting cells^34–36^. Spi-B, which is an important mediator of both early T cell lineage differentiation and B cell receptor signaling and plays a role in IL7R-induced CD40 expression, was also identified as a direct target of POU2AF1^37–40^. Furthermore, *pou2af1*^-/-^ mice failed to form germinal centers following challenge with a T cell-dependent antigen due to loss of binding to octamer-containing promoter 2 (P2) of Spi-B, which is essential for germinal center formation and maintenance^39^. Additionally, defects in B cell development and immune responses were reported in *pou2af1*^-/-^ mice^41^. Therefore, *POU2AF1,* which was a “mapped gene” at this locus, tends to be considered as the “effector gene,” which was directly regulated by the primary functional variant at this locus. In the present study, although rs1944919 was significantly affected with *POU2AF1* expression in e-QTL analysis (Fig. 2), rs1944919 did not directly regulate *POU2AF1* expression in the genotype knock-in versions of cell lines constructed using the CRISPR/Cas9 system (Fig. 3). This contradiction in e-QTL analysis was probably caused by LD between rs1944919 and other unknown variation that directly regulates *POU2AF1* expression without conferring significant PBC susceptibility. E-QTL analysis is a powerful and efficient approach for the speculation of the function of disease susceptibility genes and variations. However, our study suggested that accurate interpretations of each variation using functional evaluations are important to avoid misrepresentation by e-QTL analysis.

**Figure 6:**
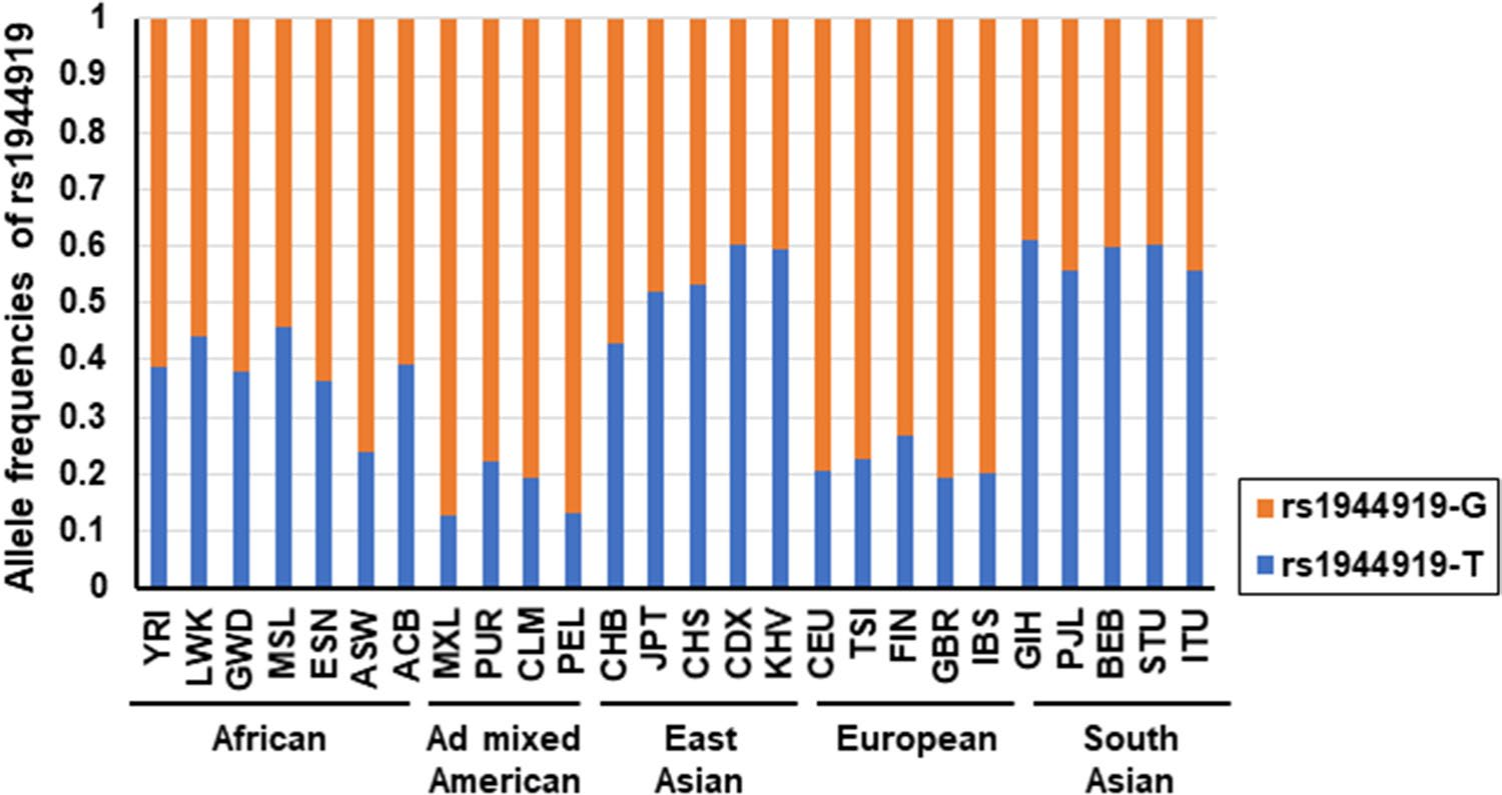
Differences in minor allele frequencies of rs1944919 among populations. The G allele is common to all populations. Frequencies of the T allele are lower in admixed American and European populations compared with other populations. YRI: Yoruba in Ibadan, Nigeria; LWK: Luhya in Webuye, Kenya; GWD: Gambian in Western Divisions in the Gambia; MSL: Mende in Sierra Leone; ESN: Esan in Nigeria; ASW: Americans of African Ancestry in Southwest USA; ACB: African Caribbean in Barbados; MXL: Mexican Ancestry in Los Angeles, CA, USA; PUR: Puerto Ricans from Puerto Rico; CLM: Colombians from Medellin, Colombia; PEL: Peruvians from Lima, Peru; CHB: Han Chinese in Beijing, China; JPT: Japanese in Tokyo, Japan; CHS: Southern Han Chinese; CDX: Chinese Dai in Xishuangbanna, China; KHV: Kinh in Ho Chi Minh City, Vietnam; CEU: Utah residents with Northern and Western European ancestry; TSI: Toscani in Italia; FIN: Finnish in Finland; GBR: British in England and Scotland; IBS: Iberian population in Spain; GIH: Gujarati Indian from Houston, TX, USA; PJL: Punjabi from Lahore, Pakistan; BEB: Bengali from Bangladesh; STU: Sri Lankan Tamil from the UK; ITU: Indian Telugu from the UK.

In the present study, rs1944919, located at the 5′ region of *POU2AF1,* was identified as the primary functional variant for PBC susceptibility. Genetic variations in chromosome 11q23.1 have been significantly associated with PBC susceptibility in the Japanese population but not in other populations^10–20^. In individuals of European descent, chromosome 11q23.1 was identified as a susceptibility gene locus for Hodgkin’s lymphoma (most significant SNP: rs7111520) and eosinophil count (most significant SNP: rs6589229, which showed r^2^ = 1 with rs7111520)^41,42^. There are several possible explanations for this difference, one of which is the variation in LD among populations. In East Asian populations (including Han Chinese in Beijing, Japanese in Tokyo, Southern Han Chinese, Chinese Dai in Xishuangbanna, and Kinh in Ho Chi Minh City), rs1944919 (i.e., the primary functional variant identified in this study) exhibited stronger LD with rs4938534 (*r*^*2*^ = 0.6237) compared with rs6589229 and rs7111520 (*r*^*2*^ = 0.3999). In contrast, in European populations (including Utah residents with Northern and Western European ancestry, Toscani in Italy, Finnish in Finland, British in England and Scotland, and Iberians in Spain), rs1944919 exhibited stronger LD with rs6589229 and rs7111520 (*r*^*2*^ = 0.516) compared with rs4938534 (*r*^*2*^ = 0.4497) (Table 2)^43^. Another possible explanation for the difference in susceptibility association is variation in the allele frequency of rs1944919 among different populations. Compared with East Asians, individuals of European descent exhibited lower minor allele frequencies (Fig. 6)^43^. An analysis covering over 3,200 cases and 3,200 controls would be needed to detect associations of rs1944919 with approximately 80% power and *P*-value threshold of 5 × 10^–8^ if rs1944919 shows the same level of OR in European descent as observed in the Japanese population^44^. These data indicate that rs1944919 might affect susceptibility to Hodgkin’s lymphoma and eosinophil count, but a GWAS with much larger sample size is necessary to determine a significant association of chromosome 11q23.1 with PBC susceptibility in individuals of European descent.

**Table 2:**
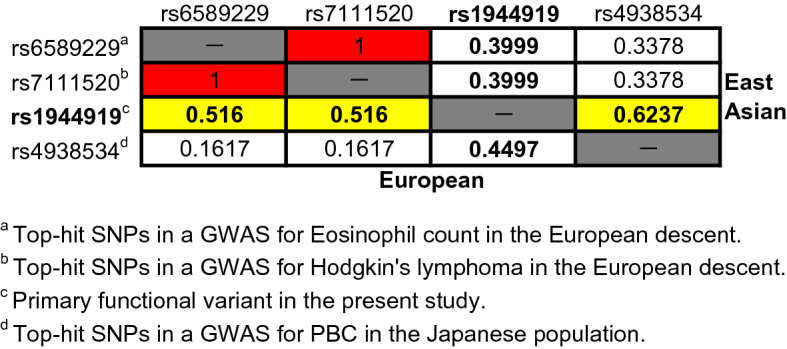
Linkage disequilibrium (r^2^) among important SNPs in chromosome 11q23.1.

^a^Top-hit SNPs in a GWAS for Eosinophil count in the European descent.

^b^Top-hit SNPs in a GWAS for Hodgkin's lymphoma in the European descent.

^c^Primary functional variant in the present study.

^d^Top-hit SNPs in a GWAS for PBC in the Japanese population.

There are some limitations to this study. Transcription factors that regulate *COLCA1* and *COLCA2* gene expression and their molecular interactions are still unknown. Additionally, molecular mechanisms of PBC susceptibility from *COLCA1*/*COLCA2* must be delineated. In order to overcome these limitations, protein expression or interaction-based studies, such as molecular biological experiments including clinical samples or experimental animal models, are needed.

In conclusion, the present high-density association mapping of chromosome 11q23.1 based on SNP imputation and in silico/in vitro functional analyses revealed the molecular mechanism by which rs1944919 increases the transcriptional efficiency of *COLCA1* and *COLCA2*. The results of the present study also demonstrated that effector genes are not always located near GWAS top-hit SNPs. Among PBC susceptibility gene loci, different “mapped gene” and “effector gene” pairs were observed on chromosome 3q13.33 and 17q12-21^20,33^. A similar systematic analysis using the methods employed in the present study would be very useful in clarifying the molecular mechanism of disease development following comprehensive genetics analyses using approaches such as GWASs.

## Materials and methods

### Subjects and research ethics

We previously described the demographic and clinical characteristics of the participants in this study^18,19^. Written informed consent was obtained from all participants. The study was approved by the Committee on Research Ethics of Hoshi University, National Center for Global Health and Medicine, and the National Hospital Organization. All methods were performed in accordance with the ethical guidelines and regulations.

### SNP imputation

A phased, whole-genome sequencing reference panel of 1,070 Japanese individuals (1KJPN) was used in this study^22^. SNP filtering and genotype imputation methods were described by Hitomi et al*.*^29^.

### Prediction tools, statistical tools, and databases

The probability that a candidate functional variant might affect transcription regulation was evaluated using the UCSC genome browser (http://genome.ucsc.edu/index.html)^23^. The allele frequency of rs1944919 in each population and linkage disequilibrium (LD) data were obtained from LDlink (https://ldlink.nci.nih.gov/)^43^. Hi-C data for chromosome 11q23.1 were obtained from the 4C browser (http://promoter.bx.psu.edu/hi-c/virtual4c.php)^28^. Statistical power was calculated using GWA power calculator (http://csg.sph.umich.edu/abecasis/gas_power_calculator/index.html)^44^. Transcription factor binding was predicted using TRANSFAC Professional (QIAGEN, Valencia, CA, USA; http://www.gene-regulation.com/pub/databases.html)^24^. *TAF1* and *EP300* gene expression levels in each cell line were determined using data available from GeneCards (http://www.genecards.org/)^25^. Correlations of all of SNPs in this locus with gene expression and splicing levels in each organ and immune cell subset were examined using data from the GTEx portal database, version 8 (http://gtexportal.org/home/), along with previously reported data (Ishigaki et al*.*)^26,27^. Expression data of *COLCA1* and *COLCA2* in lymphocytes from healthy individuals were obtained from DICE (https://dice-database.org/)^31^.

### Electrophoretic mobility shift assay (EMSA)

According to the manufacturer’s instructions, EMSA was performed using the LightShift Chemiluminescent EMSA kit (Thermo Fisher Scientific, Waltham, MA, USA) and biotinated double-strand oligonucleotide probes which were corresponded to each major and minor allele (Supplementary Table 1). 10 fmol/μL of oligonucleotide probes were incubated with 2.5 μg/mL of nuclear extract of HepG2 or Jurkat cells (Nuclear Extract Kit; Active Region, Carlsbad, CA, USA) for 30 min at 25 °C.

### Luciferase reporter assay

Human genomic DNA sequences surrounding candidate SNPs (rs1944919, rs4938534, rs7952497, rs6589227, rs6589226, and rs4356268) at the 5′ end of *POU2AF1* were amplified using specific PCR primers. (Supplementary Table 2). Amplicons made by PCR were subcloned into the pGL4.23 (luc2/minP) luciferase reporter vector (Promega, Madison, WI, USA). 500 ng of pGL4.23 constructs of each allele and 50 ng of pGL4.74 (hRluc/TK) vector as an internal control were transfected into Jurkat, HepG2, and Raji cells using Lipofectamine 3000 (Thermo Fisher Scientific). Using the Dual-Luciferase Reporter Assay system (Promega), luciferase activity was measured. Relative luciferase activity between major and minor alleles of each SNP were compared using Student’s *t* test (statistically significant level: *P* ≤ 0.05). Data in each figure represent the mean ± standard deviation of triplicate assays in a single experiment.

### Gene editing using CRISPR/Cas9

gRNA target sequences (Supplementary Table 3) were subcloned into the pGuide-it-ZsGreen1 vector (Clontech Laboratories, Mountain View, CA, USA) following the manufacturer’s protocol. pGuide-it-ZsGreen1 constructs of each target sequence and donor DNA for each allele were transfected into Jurkat and Raji cells using Lipofectamine 3000 (Thermo Fisher Scientific). Transfected clones were isolated from bulk transfectant by the BD FACS Aria II cell sorter (BD Biosciences, Franklin Lakes, NJ, USA). After single-cell cloning and extraction of genomic DNA using the PureLink Genomic DNA Mini Kit (Thermo Fisher Scientific), gene editing of target sites was checked by Sanger sequencing (ABI prism 3730-XL, Thermo Fisher Scientific) using primer sets shown in Supplementary Table 3. All knock-in clones that were derived from gene editing using CRISPR-Cas9 were used for quantitative RT-PCR analyses.

### Quantitative reverse-transcription PCR

The methods of quantitative reverse-transcription PCR were previously described^20,29^. For Raji and Jurkat clones, total RNA was extracted using the RNeasy kit (Qiagen) and first-strand complementary DNA was synthesized using the High-Capacity Complementary DNA Reverse-Transcription Kit (Thermo Fisher Scientific). Reverse-transcription PCR to detect each transcript was performed using primers shown in Supplementary Table 4 and FastStart Taq polymerase (Sigma-Aldrich, St. Louis, MO, USA). To achieve linear amplification, 21, 27, 29, and 31 cycles were found to be optimal in preliminary experiments for *GAPDH*, *POU2AF1*, *COLCA1*, and *COLCA2*, respectively. Quantitation of each transcript was performed using the Agilent 2100 Bioanalyzer (Agilent Technologies, Palo Alto, CA, USA). These experiments were repeated 3 times with essentially identical results.

## Supplementary Information

Supplementary Information
