# Supplementary material for: rs1944919 on chromosome 11q23.1 and its effector genes COLCA1/COLCA2 confer susceptibility to primary biliary cholangitis
Source: Sci Rep. 2021 Feb 25;11:4557. doi: 10.1038/s41598-021-84042-x (PMC7907150; doi:10.1038/s41598-021-84042-x)
Supplement: Supplementary file 1 — Supplementary Information [file 41598_2021_84042_MOESM1_ESM.pdf]

Supplementary data to:

# rs1944919 on chromosome 11q23.1 and its effector genes *COLCA1/COLCA2* confer susceptibility to primary biliary cholangitis

Yuki Hitomi[1,*], Yoshihiro Aiba[2], Yosuke Kawai[3], Kaname Kojima[4], Kazuko Ueno[3], Nao Nishida[3,5], Minae Kawashima[6], Olivier Gervais[7], Seik-Soon Khor[3], Masao Nagasaki[7], Katsushi Tokunaga[3], Minoru Nakamura[2,8,9], Makoto Tsuiji[1,*]

[1] Department of Microbiology, Hoshi University School of Pharmacy and Pharmaceutical Sciences, Tokyo, Japan

[2] Clinical Research Center, National Hospital Organization (NHO) Nagasaki Medical Center, Omura, Japan

[3] Genome Medical Science Project, National Center for Global Health and Medicine, Tokyo, Japan

[4] Tohoku Medical Megabank Organization, Tohoku University, Sendai, Japan

[5] The Research Center for Hepatitis and Immunology, National Center for Global Health and Medicine, Ichikawa, Japan

[6] Japan Science and Technology Agency (JST), Tokyo, Japan

[7] Human Biosciences Unit for the Top Global Course Center for the Promotion of Interdisciplinary Education and Research, Kyoto University, Kyoto, Japan

[8] Department of Hepatology, Nagasaki University Graduate School of Biomedical Sciences, Omura, Japan

[9] Headquarters of PBC Research in NHO Study Group for Liver Disease in Japan

(NHOSLJ), Clinical Research Center, NHO Nagasaki Medical Center, Omura, Japan

*Correspondence should be addressed to:

- Yuki Hitomi, PhD

  Hoshi University School of Pharmacy and Pharmaceutical Sciences

  2-4-41 Ebara, Shinagawa-ku, Tokyo 142-8501, Japan

  Tel: +81-3-5498-5755; Fax: +81-3-5498-5755

  E-mail: yhitomi-tky@umin.ac.jp

- Makoto Tsuiji, PhD

  Hoshi University School of Pharmacy and Pharmaceutical Sciences

  2-4-41 Ebara, Shinagawa-ku, Tokyo 142-8501, Japan

  Tel: +81-3-5498-5902; Fax: +81-3-5498-5755

  E-mail: m-tsuiji@hoshi.ac.jp

**Table of contents**

Supplementary Tables ....................................................................3

Supplementary Figures ..................................................................7

## Supplementary Tables

**Table S1.** Oligo-nucleotide probes for the EMSA

| SNP | Allele | Sequence (5' -> 3') |
|---|---|---|
| rs4938534 | T | TCCTCTTTCCCGACGTCGGGTAGTTTCTTAA |
| | C | TCCTCTTTCCCGACGCCGGGTAGTTTCTTAA |
| rs7952497 | C | CCATCCCCCTTCCCCCGTTACAAAAAGGACA |
| | A | CCATCCCCCTTCCCCAGTTACAAAAAGGACA |
| rs6589227 | T | TCCTGTGCGAAGTGGTTCGGTCTAGAGGTGA |
| | C | TCCTGTGCGAAGTGGCTCGGTCTAGAGGTGA |
| rs1944919 | T | TCGAGGTGGGTACTGTCTTTCAACGGGTCCT |
| | G | TCGAGGTGGGTACTGGCTTTCAACGGGTCCT |
| rs6589226 | G | TTCAAGTGTTAACAAGGTAGGTTGAGGCCCC |
| | A | TTCAAGTGTTAACAAAGTAGGTTGAGGCCCC |
| rs4356268 | T | CCGATACGGGTGCCTTTTTCTCCGGTTGGGA |
| | C | CCGATACGGGTGCCTCTTTCTCCGGTTGGGA |

**Table S2.** Primers for production of Luciferase assay constructs.

| Primer Name | Sequence (5' -> 3') |
|---|---|
| rs1944919-F | GATATCTTTGCATGCATGCCATGCTC |
| rs1944919-R | AGATCTGAAAAACAGCAGTGCAGCTG |
| rs4938534-F | GGTACCTTCATGGTGTGCTTCACCTC |
| rs4938534-R | GAGCTCGCCTCGGGTATGTCTTTATC |
| rs7952497-F | GGTACCGCTTAAAACTGTGGAGGGAC |
| rs7952497-R | GAGCTCACATAGGAGCCCAATGAGAC |
| rs6589227-F | GGTACCCCTGTTTCTAGCTGAGTGAC |
| rs6589227-R | GAGCTCTTTCATCCAACTCCGGGGAT |
| rs6589226-F | GGTACCCTTCTGTGACAAGGCTTGTC |
| rs6589226-R | GAGCTCGGTTCAACTCTCAGAAGCTG |
| rs4356268-F | GGTACCGAGGCTCTGTTCATCTTGTG |
| rs4356268-R | GAGCTCTTGAAACACAGTAGCCCCTC |

**Table S3:** Sequences of primers used for the synthesis of gRNAs, donor-DNA, and sequence check used for gene editing.

| gRNA/donor DNA | Sequence |
|---|---|
| gRNA-1 primer-F | CCGGCCATGCTCCTGGGCAACTTT |
| gRNA-1 primer-R | AAACAAAGTTGCCCAGGAGCATGG |
| gRNA-2 primer-F | CCGGTCCTGGGCAACTTTCGGTCA |
| gRNA-2 primer-R | AAACTGACCGAAAGTTGCCCAGGA |
| gRNA-3 primer-F | CCGGTCCTGGGCAACTTTCTGTCA |
| gRNA-3 primer-R | AAACTGACAGAAAGTTGCCCAGGA |
| donor ssDNA-G | AACAGCAGTGCAGCTGCCAGCCCTATAGCT<br>CCACACATGACCGAAAGTTGCCCAGGAGCA<br>TGGCATGCATGCAAATAGACT |
| donor ssDNA-T | AACAGCAGTGCAGCTGCCAGCCCTATAGCT<br>CCACACATGACAGAAAGTTGCCCAGGAGCA<br>TGGCATGCATGCAAATAGACT |
| Sequence check-F | CAGGAGTTTCACCAGGTGTA |
| Sequence check-R | GAAAAACAGCAGTGCAGCTG |

**Table S4:** Sequences of primers used for quantitative RT-PCR.

| Gene Function | Gene name | amplicon size | Primer name | Sequence |
|---|---|---|---|---|
| Candidate effector gene | *POU2AF1* | 71 bp | POU2AF1-F | TGGCGACCTACACCACAGT |
| | | | POU2AF1-R | CTCCTCTGTCACTGCAGAC |
| | *COLCA1* | 155 bp | COLCA1-F | ATGTGGAGATGGACAGGGAT |
| | | | COLCA1-R | TCCTTGCTGTCCTTACAGAC |
| | *COLCA2* | 113 bp | COLCA2-F | TCCGAGTGAAGATCACAGTG |
| | | | COLCA2-R | TGCAACTGGGTCTGAAAGGT |
| Housekeeping | *GAPHD* | 194 bp | GAPDH-F | GCCAAGGTCATCCATGACAA |
| | | | GAPDH-R | TTCAGCTCAGGGATGACCTT |

## Supplementary Figures

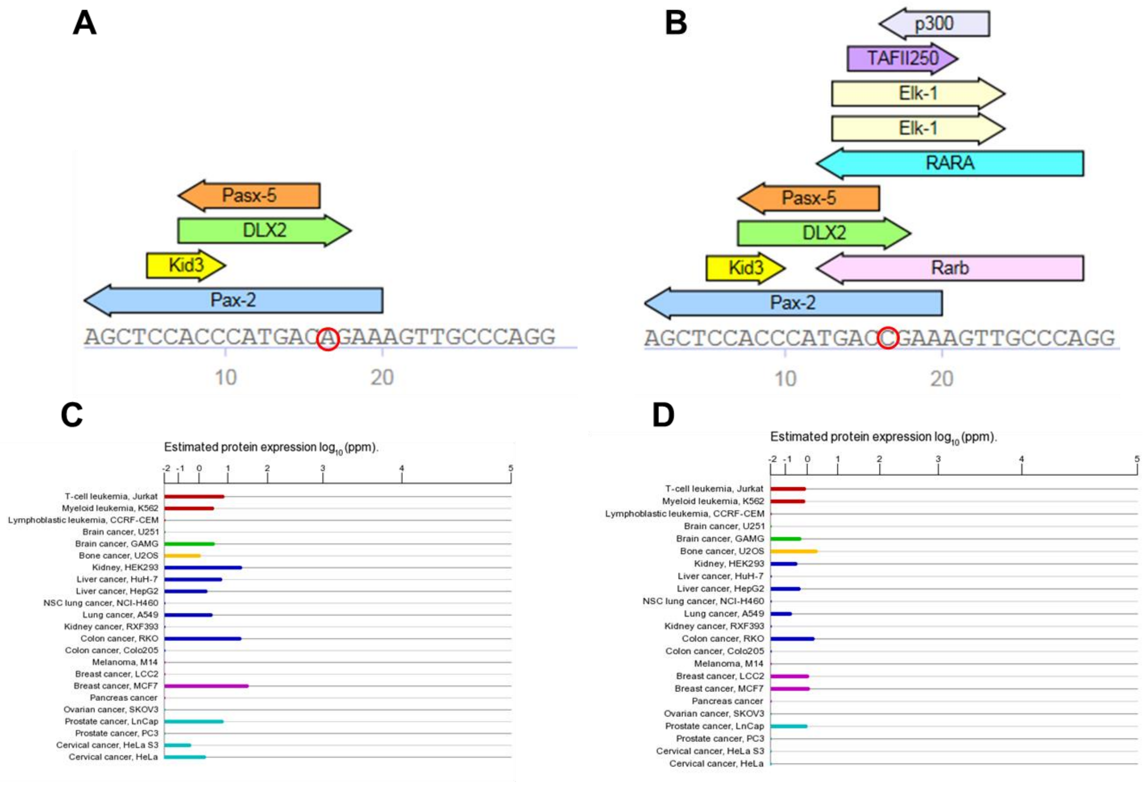


**Supplementary Fig. 1. Prediction of transcription factor binding using the TRANSFAC database. (A)** Predicted transcription factor binding in T allele of rs1944919. Reverse complement DNA sequences are shown. **(B)** Predicted transcription factor binding in G allele of rs1944919. Several transcription factors bind to G allele compared with T allele **(B)**. **(C and D)** EP300 **(C)** and TAF2 **(D)** expression levels in cancer cell lines. Both Jurkat and HepG2 cells showed abundant EP300 and TAF2 expression. Expression data were obtained using GeneCards from the Weizmann Institute of Science (http://www.genecards.org/).

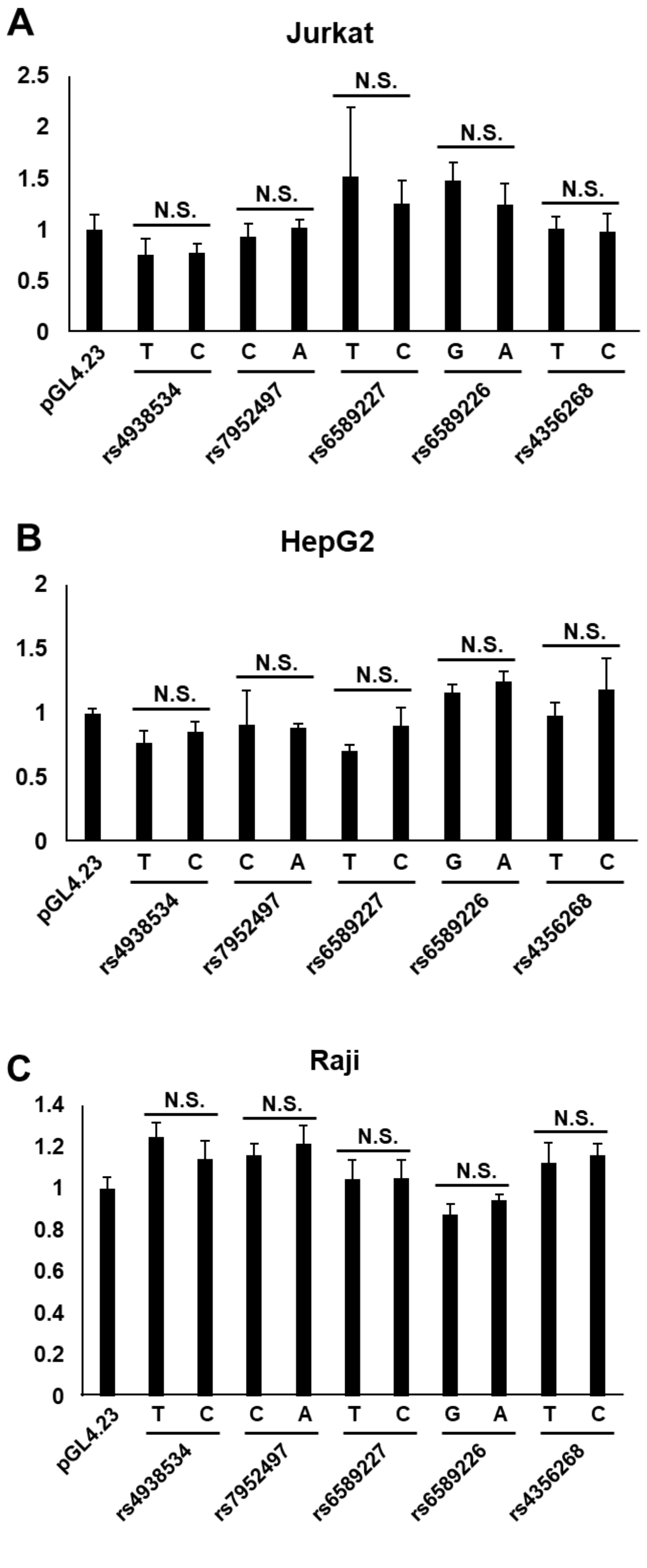


**Supplementary Fig. 2. Luciferase assay for other candidate SNPs.**

Transcription was measured by cellular luciferase activity 24 h after transfection of Jurkat **(A),** HepG2 **(B),** and Raji **(C)** cells. No differences in luciferase activity were observed for the other candidate SNPs. Three independent experiments with triplicate measurements were performed for each assay. Data represent the mean ± SD; “N.S.” means non-significant (Student’s *t* test).

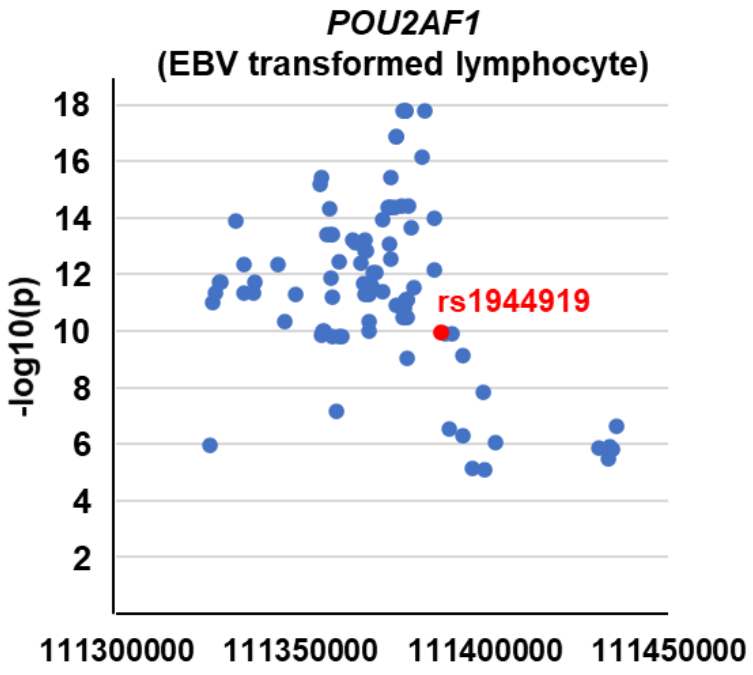


**Supplementary Fig. 3. s-QTL mapping of *POU2AF1* in the EBV transformed lymphocyte.** SNPs whose P-values less than $1.0 \times 10^{-4}$ were shown. rs1944919, shown as red solid dots, did not exhibit the strongest association with *POU2AF1* splicing. rs4938534, which was the most significant SNP with PBC susceptibility on chromosome 11q23.1, did not associated with *POU2AF1* splicing ($P > 1.0 \times 10^{-4}$)
